# Internal vertebral morphology of bony fishes matches the mechanical demands of different environments

**DOI:** 10.1002/ece3.9499

**Published:** 2022-11-18

**Authors:** Dana Baxter, Karly E. Cohen, Cassandra M. Donatelli, Eric D. Tytell

**Affiliations:** ^1^ Department of Biology Tufts University Medford Massachusetts USA; ^2^ Department of Biology University of Florida Gainesville Florida USA; ^3^ Friday Harbor Laboratories, University of Washington Friday Harbor Washington USA; ^4^ Fowler School of Engineering Chapman University Orange California USA

**Keywords:** mechanics, statistical modeling, vertebral morphology

## Abstract

Fishes have repeatedly evolved characteristic body shapes depending on how close they live to the substrate. Pelagic fishes live in open water and typically have narrow, streamlined body shapes; benthic and demersal fishes live close to the substrate; and demersal fishes often have deeper bodies. These shape differences are often associated with behavioral differences: pelagic fishes swim nearly constantly, demersal fishes tend to maneuver near the substrate, and benthic fishes often lie in wait on the substrate. We hypothesized that these morphological and behavioral differences would be reflected in the mechanical properties of the body, and specifically in vertebral column stiffness, because it is an attachment point for the locomotor musculature and a central axis for body bending. The vertebrae of bony fishes are composed of two cones connected by a foramen, which is filled by the notochord. Since the notochord is more flexible than bony vertebral centra, we predicted that pelagic fishes would have narrower foramina or shallower cones, leading to less notochordal material and a stiffer vertebral column which might support continuous swimming. In contrast, we predicted that benthic and demersal fishes would have more notochordal material, making the vertebral column more flexible for diverse behaviors in these species. We therefore examined vertebral morphology in 79 species using micro‐computed tomography scans. Six vertebral features were measured including notochordal foramen diameter, centrum body length, and the cone angles and diameters for the anterior and posterior vertebral cones, along with body fineness. Using phylogenetic generalized least squares analyses, we found that benthic and pelagic species differed significantly, with larger foramina, shorter centra, and larger cones in benthic species. Thus, morphological differences in the internal shape of the vertebrae of fishes are consistent with a stiffer vertebral column in pelagic fishes and with a more flexible vertebral column in benthic species.

## Graphical abstract

The internal morphology of fish vertebral centra differs for fish from benthic, demersal, or pelagic habitats. Pelagic fishes have smaller cones and narrower vertebral foramina than species from the other habitats, reducing the amount of flexible notochordal tissue between their vertebrae. Pelagic fishes therefore likely have stiffer vertebral columns, which may be important functionally to support more continuous swimming.

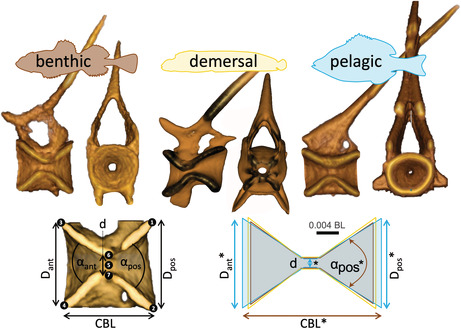

## 1 INTRODUCTION

Many groups of fishes have evolved specialized forms for living close to the bottom of a body of water, called a benthic habitat, and for living in open water, a pelagic habitat (Burress et al., 2017; Friedman et al., 2020; Hollingsworth et al., 2013; Hulsey et al., 2013; Kusche et al., 2014; Ribeiro et al., 2018; Robinson & Wilson, 1994; Willacker et al., 2010). In benthic habitats, fishes spend much of their time in contact with the substrate and often use “lie‐and‐wait” predation strategies (e.g., flatfishes: Link et al., 2002). In pelagic habitats, fishes usually swim constantly and often evolve an elongate, streamlined form (Friedman et al., 2020; Tavera et al., 2018) that is thought to be advantageous for steady swimming (Lauder, 2015). Often, another category is added, termed demersal. In these habitats, fishes live in close proximity to the substrate, but do not typically sit directly on it. They often feed by sifting detritus through sand or scraping algae off of coral and rocks and tend to have deeper bodies (Friedman et al., 2020; Larouche et al., 2020).

This diversification in body shape across habitat categories was most thoroughly evaluated recently by Friedman et al. (2020). Across a large sample of fish species, they found subtle, but significant differences in body shape, particularly in benthic fishes. Compared to benthic fishes, pelagic and demersal fishes tended to have deeper bodies and a narrower range of body widths. Benthic fishes include both flatfishes, with extremely high body width, and elongate fishes, with more narrow bodies. Indeed, benthic fishes had the largest diversity of body shapes associated with the highest rate of body shape evolution (Friedman et al., 2020).

Within individual clades, the pattern of differences in species from benthic, demersal, and pelagic habitats is often present and can be even more pronounced than Friedman et al. (2020) found when considering many taxa. For example, cichlid species in the African Rift Lakes have repeatedly and convergently evolved streamlined shallow‐bodied pelagic forms and deep‐bodied benthic and demersal forms (Cooper et al., 2010; Muschick et al., 2012). Similar patterns have been seen in many different groups of fishes, including grunts (Tavera et al., 2018), new world cichlids (Kusche et al., 2014), cyprinids (Hollingsworth et al., 2013), and in Carangaria, a large group of marine fishes that includes both flatfishes, an extreme benthic morphology, and billfishes, an extreme pelagic morphology (Ribeiro et al., 2018).

These morphological shifts can be observed even in individual species or genera, where their functional consequences are clearer. Within three‐spine stickleback species *Gasterosteus* spp., shallower and deeper bodied ecomorphs have evolved multiple times in separate lakes. Ecomorphs that primarily feed on benthic prey and tend to stay close to the substrate (“demersal” in the classification we use) have deeper bodies, while those that feed in open water (“pelagic”) have more streamlined bodies (Schluter, 1993; Walker, 1997; Willacker et al., 2010). The pelagic ecomorphs had lower drag coefficients and could swim faster for longer than the demersal species (Blake et al., 2005). Similarly, bluegill sunfish (*Lepomis macrochirus*) have pelagic and demersal ecomorphs, often in the same lakes, where the pelagic morphs have more streamlined bodies and demersal morphs had deeper and wider bodies (Ehlinger & Wilson, 2006; Gerry et al., 2011). These morphological differences correspond to lower cost of transport during steady swimming in pelagic morphs (Ellerby & Gerry, 2011) and faster turning performance in demersal morphs (Gerry et al., 2012).

In this extensive body of literature, we see a consistent pattern in which pelagic species have more streamlined bodies and swim more constantly and at higher speeds than demersal and benthic species. In contrast, demersal and benthic species often have deeper bodies and greater turning performance than pelagic species. We hypothesized that the external morphological differences would be accompanied by internal differences. In particular, since these differences are related to locomotion, which is driven by the axial musculature that acts to bend the vertebral column, we hypothesized that they might be accompanied by differences in the morphology of the vertebral column. The vertebral column both serves to resist bending forces from the axial musculature and as an attachment point for those muscles. Overall body stiffness is thought to contribute to high speed, continuous swimming (Koob & Long, 2000; Summers & Long, 2006); we therefore predicted that pelagic species that swim continuously should have a morphology associated with a stiffer vertebral column than demersal or benthic species. Benthic and demersal species, in contrast, are often “lie‐and‐wait” predators (e.g., flatfishes: Link et al., 2002), attacking prey with rapid accelerations that require high body curvature (Akanyeti et al., 2017; Schwalbe et al., 2019).

Donatelli et al. (2021) recently examined how the internal shape of vertebrae in fishes affects the stiffness of the vertebral column. In particular, they showed that the shape of the intervertebral joints, which are filled by the notochord, has a substantial effect on the stiffness of the vertebral column. Teleost fishes have characteristically hourglass‐shaped vertebral centra, called amphicoelous centra, consisting of anterior and posterior cones, joined at the tips (Laerm, 1976). The notochordal tissues (i.e., the notochordal cell mass, notochordal epithelium, and notochordal strand) fill the inside of the centra, including a hole through the middle called the notochordal foramen, and makes up most of the intervertebral joint (Symmons, 1979). The bony elements of the centra are joined via a complex of soft tissues (the encapsulating complex) including the external intervertebral ligament (EVL), elastica externa (EE), and a fibrous sheath (FS) (Symmons, 1979). Donatelli et al. (2021) found that vertebral segments with larger foramina and larger cone angles tended to be more flexible. Overall, the mechanical properties of the joints contribute to the stiffness of the vertebral column (Long, 1992; Long et al., 2004; Nowroozi & Brainerd, 2012, 2014; Porter & Long, 2010), although others have questioned whether fish bend enough during normal swimming for the intervertebral joints to have any effect (Nowroozi & Brainerd, 2013).

We therefore examined the variation in internal vertebral morphology in benthic, demersal, and pelagic fishes across actinopterygian fishes. To identify differences in vertebral morphology of these fishes due to habitat, we controlled for shared evolutionary history using the Rabosky et al. (2018) phylogenetic tree. Based on our hypothesis that internal morphological differences support behavioral differences across different habitat groups, we predicted that pelagic fishes should have a more closed internal vertebral morphology minimizing space for soft material and leading to a stiffer backbone, and that benthic and demersal species should have a morphology that allows for more flexibility in both mechanics and behavior.

## 2 METHODS

**FIGURE 1:**
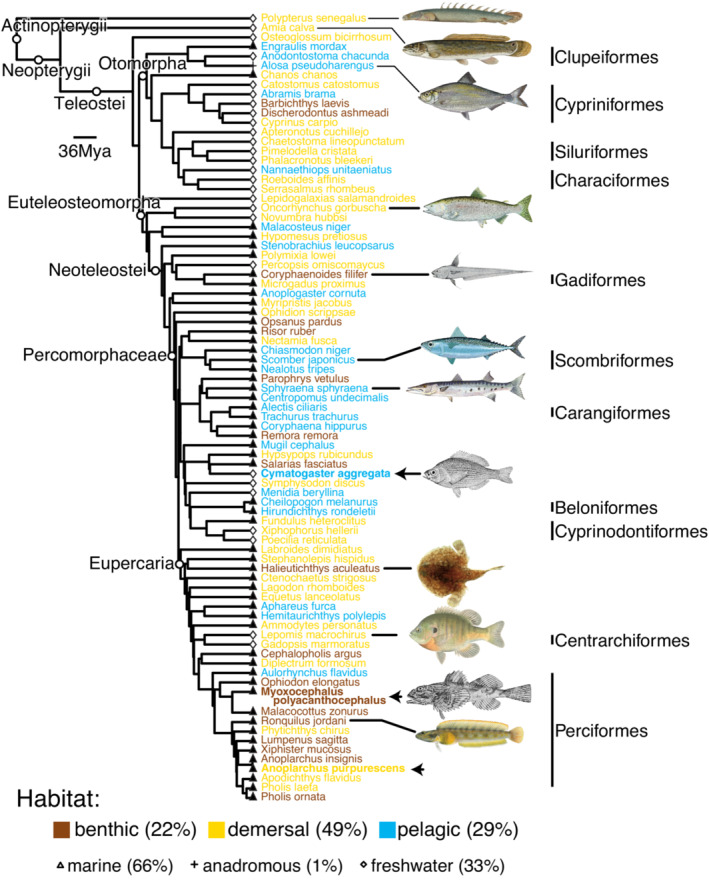
Phylogenetic tree colored by habitat category. Phylogeny (trimmed from Rabosky et al., 2018) displaying the 79 species included in this study, with color indicating habitat and shapes of points indicating marine or freshwater species. Example species with histological analysis are identified with arrows (see Figure 6). Order names are given on the right where we have multiple species in the same order, and higher level taxonomic groups are shown with open circles. All images are from the public domain, with sources given in Table S1.

We used micro‐computed tomography (μCT) scans to measure six different vertebral features from 79 species across a trimmed version of the Rabosky et al. (2018) phylogeny (Figure 1). We classified these species into benthic (species in contact with the substrate most of the time), pelagic (open water swimmers), and demersal (species close to the substrate but not resting on it all of the time) habitats. For all of the species we examined that overlapped those from Friedman et al. (2020), we used their classification. For other species, we classified habitat based on descriptions of each species from guidebooks or relevant journal articles (Allen et al., 2002; Bailey, 1994; Basolo, 1990; Fine et al., 1987; Gilbert & Williams, 2002; Jaafar et al., 2004; Lowry et al., 2005; Magid, 1967; Matsui & Rosenblatt, 1987; McGinnis & Alcorn, 2006; Mérigoux et al., 1998; Page et al., 1991; Pearcy et al., 1982; Phomikong et al., 2015; Pietsch & Orr, 2015; Proctor & Lynch, 2011; Rodríguez‐Olarte et al., 2011; van der Sleen & Albert, 2018; Vašek et al., 2008).

We used histological data to compare the soft tissue anatomy across representative species from benthic, pelagic, and demersal habitats. No animals were sacrificed for histological analyses. Instead, all samples were donated from the Friday Harbor Labs Ichthyology collection. All specimens arrived fixed in 10% buffered formalin solution and stored in 70% ethanol (EtOH). The vertebral columns from *Anoplarchus purpurascens* (prickleback, demersal), *Cymatogaster aggregata* (perch, pelagic), and *Myoxocephalus polyacanthocephalus* (sculpin, benthic) were carefully dissected and three full vertebrae near 50% total length were removed. These vertebrae were chosen as they are the most representative of each species' vertebral morphology and not as influenced by other intrinsic factors such as head or tail shape. The vertebrae were decalcified in a 10% EDTA solution for 10 days. After full decalcification, vertebrae were rinsed with dH_2_O for 48 h before undergoing an ethanol dehydration series to 95% EtOH. Each sample was then infiltrated and embedded with JB4 embedding media (14272‐00) following Electron Microscopy Sciences JB4 embedding media protocol. Each species was sectioned at thicknesses between 3 and 3.5 μm with a glass knife, stained with Lee's Basic Fuchsin and Methylene Blue, and mounted to glass slides with permount. We imaged slides on a Nikon Eclipse E600 microscope with a Micropublisher 5.0 RTV camera. Whole slice histological images were tiled and stitched together and color balanced in Adobe Photoshop. This method is a composite that allows for high‐resolution images to be taken of large histological slices while still viewing the whole section.

### 2.1 Vertebral measurements

Most of the μCT scans were downloaded as image stacks off of Morphosource (https://www.morphosource.org/) or the “CT Scans ‐ #ScanAllFish” (Adam Summers, https://osf.io/ecmz4/) database. Additional specimens were scanned at the Karel F. Liem Bioimaging facility using their Bruker Skyscan 1173 (Bruker microCT). Full identification of all scans is given in Table S1.

**FIGURE 2:**
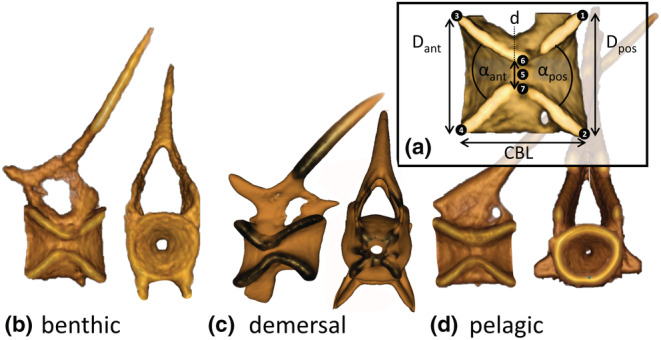
Vertebral centra measurements. (a) Measurements taken from vertebral centra, based on the numbered points. (a) *d* is foramen diameter, CBL is centrum body length, $\alpha_{\mathrm{ant}}$ is anterior cone angle, $D_{\mathrm{ant}}$ is anterior cone diameter, $\alpha_{\mathrm{pos}}$ is posterior cone angle, $D_{\mathrm{pos}}$ is posterior cone diameter. See Table 1 for formulas. (b–d) Rendering of vertebrae in lateral and frontal views from three representative species. Lateral views show are vertebrae bisected down the middle along the sagittal plane. (b) *Myoxocephalus polyacanthocephalus* (benthic), (c) *Lumpenus sagitta* (demersal), (d) *Cymatogaster aggregata* (pelagic).

We measured vertebral features for vertebrae at 0.4–0.9 of the fish's standard length (BL), in increments of 0.1. We first measured the location of the snout and the final vertebra before the caudal fin to estimate standard length, then used that to calculate the locations at 0.4–0.9 BL. We then placed landmarks at seven points on sagittal sections through the center of vertebrae at each location if a vertebral centrum was present (Figure 2a). Based on those points, we computed the anterior and posterior cone diameter and angle, the notochordal foramen diameter, and the centrum body length (CBL) (formulas in Table 1). We also estimated fineness by dividing standard length by the maximum body width. All linear measurements were normalized to body length by dividing by the fish's standard length before we ran our statistical analyses.

**TABLE 1:** Summary of formulas for the vertebral morphology variables (Figure 2a)

Variable	Symbol	Formula
Notochordal foramen diameter	d	$\left\|P_{6}-P_{7}\right\|$
Centrum body length	CBL	$\frac{1}{2}\left(\left|P_{1,x}-P_{3,x}\right|+\left|P_{2,x}-P_{4,x}\right|\right)$
Anterior cone angle	$\alpha_{\mathrm{ant}}$	$\frac{{\left\|P_{5}-P_{4}\right\|}^{2}+{\left\|P_{5}-P_{3}\right\|}^{2}+{\left\|P_{3}-P_{4}\right\|}^{2}}{2\left\|P_{5}-P_{4}\right\|\left\|P_{5}-P_{3}\right\|}$
Anterior cone diameter	$D_{\mathrm{ant}}$	$\left\|P_{3}-P_{4}\right\|$
Posterior cone angle	$\alpha_{\mathrm{pos}}$	$\frac{{\left\|P_{5}-P_{2}\right\|}^{2}+{\left\|P_{5}-P_{1}\right\|}^{2}+{\left\|P_{1}-P_{2}\right\|}^{2}}{2\left\|P_{5}-P_{2}\right\|\left\|P_{5}-P_{1}\right\|}$
Posterior cone diameter	$D_{\mathrm{pos}}$	$\left\|P_{1}-P_{2}\right\|$

*Note*: $P_{i}$, the $\left(x,y\right)$ coordinates of the ith point on each vertebra (Figure 2a). $\left\|x\right\|$, length of the vector x.

### 2.2 Statistical analysis

To examine differences in vertebral morphological measurements as they relate to pelagic, demersal, or benthic habitat categories, as well as to control for shared evolutionary history, we performed phylogenetic generalized least squares analyses (Adams & Collyer, 2018), with the phylogenetic effect modeled using a random Brownian correlation.

We first normalized the *x*,*y* coordinates of each point ($P_{1}-P_{7}$) for each vertebra (42 points in total) by subtracting the centroid of each individual vertebra, then dividing the coordinates by the standard length of each specimen. We aligned the coordinates using a generalized Procrustes analysis (Gower, 1975), followed by a multivariate phylogenetic generalized least squares analysis using a residual randomization procedure (Collyer & Adams, 2018) to identify differences relative to habitat and fineness. Following the initial multivariate test, we also tested the coordinates for each vertebra individually (seven points per vertebra), to examine differences along the body. Because fineness varies among habitats, we were not able to include an interaction effect between habitat and fineness in our statistical models.

We then computed the six shape parameters (Table 1) and took their mean values across all vertebrae for each species. We used a multivariate PGLS analysis to compare all the parameters simultaneously, then ran univariate tests on each parameter individually.

If we found that the habitat effect was significant, we then ran the same PGLS model on each pair of habitats separately to examine the pairwise differences. In this case, we controlled for multiple comparisons with a Bonferroni correction.

We fit the models using R (version 4.2.0; https://www.R‐project.org/) with packages geomorph (version 4.0.4; Baken et al., 2021) and RRPP (version 1.3.0; Collyer & Adams, 2018). All code and data are available with DOI: 10.25833/772t‐cw09 and on Github (https://tytell.github.io/BaxterVertEvol/). The main data table is available in Table S1.

## 3 RESULTS

We examined 79 species of actinopterygian fishes based on publicly available CT scans, aiming to cover as many families as possible across the phylogeny. These species thus represented 68 different families. We classified the species' habitats as benthic (22% of species in our data set), demersal (49%), or pelagic (29%). Marine species made up 66% of the data set, and freshwater species were 33%, and we had one anadromous species.

### 3.1 Mean morphology of vertebrae differ in fishes from different habitats

**FIGURE 3:**
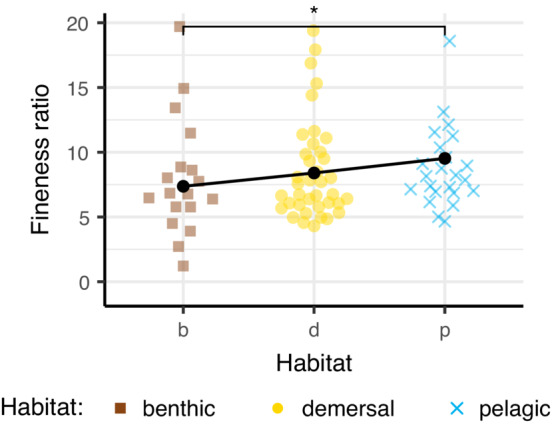
Pelagic fishes tend to be more elongate than benthic fishes. Fineness ratio relative to habitat, where larger numbers indicate more elongate fishes. Black points represent means estimated from the PGLS model. *p < .05.

Species from different habitats had different fineness (Figure 3; *p* = .048). Pelagic species tended to be more elongate (higher fineness ratio) than benthic species, though, due to the Bonferroni correction, this difference was not significant.

Next, we compared the landmarks for all digitized points for all vertebrae (42 points for each species), using a generalized Procrustes analysis to align the points, and a multivariate residual randomization procedure to compare them in a phylogenetic context (Baken et al., 2021; Collyer & Adams, 2018). We measured vertebrae at positions from 0.4 to 0.9 BL in steps of 0.1 BL (six positions per species). Species from different habitats had significantly different vertebral morphology (*p* = .045; Table 2). Fineness was not significant (*p* = .3).

**TABLE 2:** Results of multivariate phylogenetic generalized least squares analyses on landmarks for differences relative to habitats and fineness.

Position (BL)	*df*	Pillai's trace	*Z*	*p*
All vertebrae^a^				
**Habitat**	**2**	**1.9**	**0.95**	**.045**
Fineness	1	0.93	0.46	.301
0.4^b^				
**Habitat**	**2**	**0.92**	**2.9**	**.001**
Fineness	1	0.079	−0.91	.826
0.5^b^				
**Habitat**	**2**	**0.96**	**2.7**	**.001**
Fineness	1	0.22	1.2	.115
0.6^b^				
**Habitat**	**2**	**0.85**	**2.7**	**.002**
Fineness	1	0.21	1.2	.135
0.7^b^				
**Habitat**	**2**	**0.95**	**2.6**	**.001**
**Fineness**	**1**	**0.30**	**1.9**	**.031**
0.8^b^				
**Habitat**	**2**	**0.94**	**2.7**	**.001**
Fineness	1	0.21	1.1	.131
0.9^b^				
**Habitat**	**2**	**0.84**	**2.6**	**.014**
Fineness	1	0.19	1.0	.168

*Note*: Significant effects are shown in bold.

^a^ *N* = 79 species, 42 points per species.

^b^ *N* = 79 species, 7 points per species.

**FIGURE 4:**
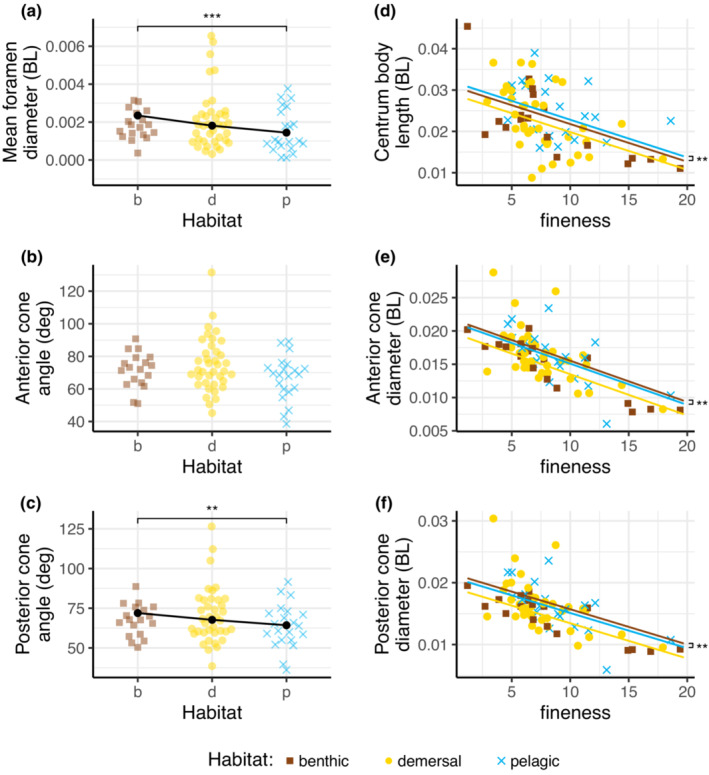
The internal shape of vertebrae is different for fishes from different habitats and with different fineness ratios. (a–c) Mean foramen diameter, anterior cone angle, and posterior cone angle showing that pelagic species have a smaller foramen and posterior cone angle than benthic species. Points are jittered left or right using a beeswarm algorithm to indicate the approximate number of species with a particular value for each parameter. Significant differences among habitats are indicated with brackets. (d–f) Mean centrum body length, anterior cone diameter, and posterior cone diameter relative to fineness ratio, showing that more elongate fishes (higher fineness) tend to have shorter vertebrae with smaller diameter cones. Significant differences among regression line intercepts are given with brackets on the right. Each colored point represents the mean across all vertebrae for a single species; black points in (a–c) show the overall means for each habitat estimated from the PGLS model; and colored lines in (d–f) show the regression lines for each habitat from the models.

Overall, we found that these mean shape parameters are different in different habitats (*p* = .001; Table 3, Figure 4) and that they also depend on the fineness ratio (*p* = .001; Table 3). The notochordal foramen diameter differed among fishes from different habitats (*p* = .001; Figure 4a; Table 4), but did not depend on fineness ratio. Pelagic fishes had smaller foramina than benthic fishes (*p* = .001). The posterior cone angle was also significantly different in fishes from different habitats (*p* = .01; Figure 4c; Table 4). Pelagic fishes had smaller posterior cone angles than benthic fishes (*p* = .005). Anterior cone angle was not significantly different in fishes in different habitats (*p* = .228; Figure 4b). Fineness ratio did not affect either cone angle significantly.

**TABLE 3:** Results of multivariate phylogenetic generalized least squares analyses for differences in overall means for each species relative to habitats and fineness.

Effect	*df*	Pillai's trace	*Z*	*p*
**Habitat**	**2**	**0.621**	**3.48**	**.001**
**Fineness**	**1**	**0.515**	**4.57**	**.001**

*Note*: Significant effects are shown in bold. Variables included are centrum body length, foramen diameter, anterior and posterior cone angles and diameters. *N* = 79 species.

**TABLE 4:** Results of phylogenetic generalized least squares analyses for differences among habitats

Effect	*df*	*r* ^2^	*F*	*Z*	*p*	Pairwise comparisons^a^		
						*p* − *b* ^b^	*d* − *b* ^c^	*p* − *d* ^d^
Fineness								
**Habitat**	**2**	**0.09**	**3.6**	**1.6**	**.048**	0.040	0.152	0.434
Centrum body length								
**Habitat**	**2**	**0.11**	**6.8**	**2.6**	**.007**	**0.011**	0.287	0.416
**Fineness**	**1**	**0.23**	**29**	**3.6**	**.001**			
Foramen diameter								
**Habitat**	**2**	**0.29**	**15**	**3.5**	**.001**	**0.001**	0.135	0.144
Fineness	1	0.03	2.9	1.4	.081			
Anterior cone angle								
Habitat	2	0.04	1.5	0.77	.228			
Fineness	1	0.00	0.0059	−1.6	.943			
Posterior cone angle								
**Habitat**	**2**	**0.11**	**4.8**	**2.2**	**.010**	**0.005**	0.254	0.471
Fineness	1	0.00	0.00028	−2.1	.989			
Anterior cone diameter								
**Habitat**	**2**	**0.22**	**20**	**3.7**	**.005**	**0.005**	0.583	0.563
**Fineness**	**1**	**0.24**	**44**	**4.0**	**.001**			
Posterior cone diameter								
**Habitat**	**2**	**0.25**	**22**	**3.6**	**.007**	**0.006**	0.515	0.725
**Fineness**	**1**	**0.19**	**34**	**3.8**	**.001**			

*Note*: Significant effects are shown in bold. *df*, degrees of freedom; *r* ^2^, a measure of the goodness of fit of the statistical model; *F*, statistical *F* parameter; *Z*, a measure of the overall effect size; *p*, probability of seeing an effect that large due to random variation.

^a^ The cutoff for a significant pairwise effect is *p* < .016 due to Bonferroni correction.

^b^ Pelagic relative to benthic.

^c^ Demersal relative to benthic.

^d^ Pelagic relative to demersal.

The CBL and anterior and posterior cone angles varied significantly among habitats (*p* < .011 in all cases; Table 4) and also decreased significantly as fineness ratio increased (Figure 4d–f). Pelagic species had significantly longer vertebrae (larger CBL) than benthic (*p* = .011), but benthic species had larger anterior and posterior cones (*p* = .005 and .006) than pelagic. These differences were small and may not be functionally relevant. Demersal species varied more than the other groups, and thus did not show any significant differences.

**FIGURE 5:**
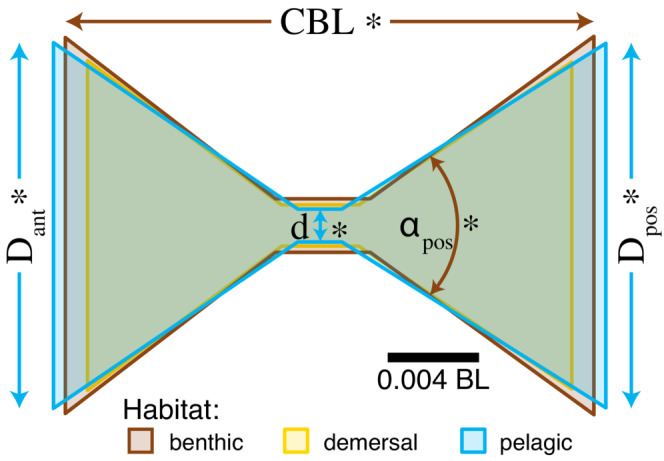
Overall mean internal shape of the vertebrae from the three habitats. The central foramen diameter (d) and the posterior cone diameter ($\alpha_{\mathrm{pos}}$) are significantly different in the different habitats, with pelagic species (blue) tending to have smaller foramina and narrower cones than the other species.

Figure 5 shows the mean shape of the internal parts of the vertebrae. Posterior cone angle ($\alpha_{\mathrm{pos}}$) and foramen diameter (d) are both significantly smaller in pelagic species, while CBL is longer in pelagic species. Both anterior and posterior cone diameters were slightly, but significantly, larger in benthic species.

### 3.2 Patterns of vertebral morphology along the body differ in fishes from different habitats

Our analysis showed that the way vertebral morphology varied along the body differed in our three habitat groups. To compare these patterns, we compared the landmarks for vertebrae at each location along the body among all of the species. We found that vertebrae at all locations were significantly different in different habitats (*p* < .039 in all cases; Table 2).

**FIGURE 6:**
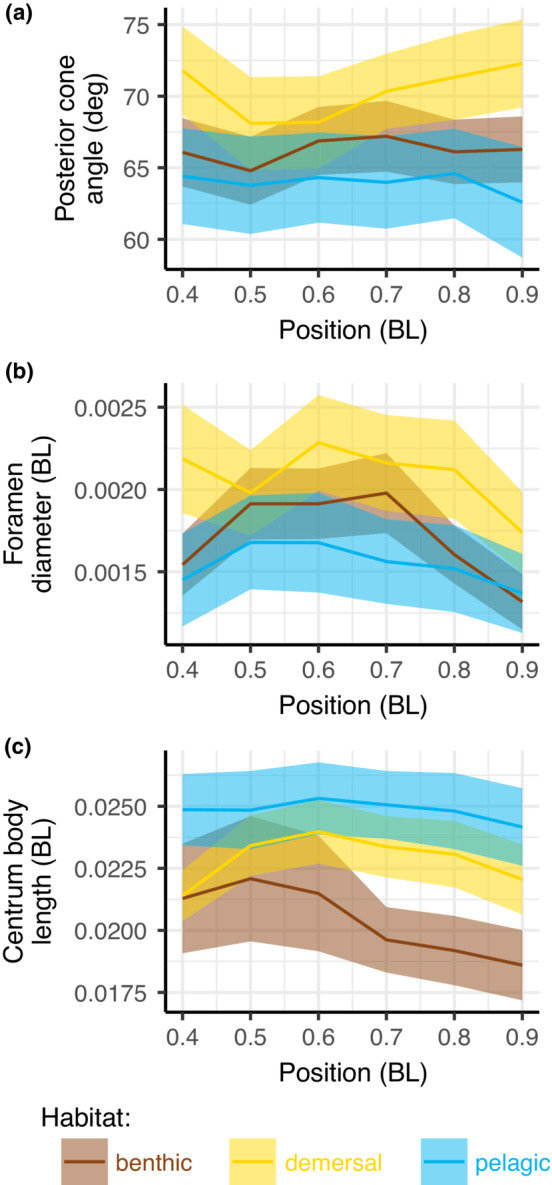
Species from different habitats differ in the distribution of vertebral shape along the body. Panels show traits where we saw significant differences among habitats. Each panel shows mean ± standard error for three parameters for vertebrae from 0.4 to 0.9 BL. (a) Posterior cone angle differs among habitats but does not vary substantially along the body. (b) Foramen diameter tends to be largest in demersal species and becomes smaller in more posterior vertebrae in all species. (c) Centrum body length becomes smaller in posterior vertebrae, particularly in benthic species.

We then calculated the same shape parameters for each vertebra individually and plotted them as a function of location. Figure 6 shows mean ± standard error for posterior cone angle, foramen diameter, and CBL relative to position along the body. Posterior cone angle does not vary substantially along the body (Figure 6a). Foramen diameter tends to be largest near the mid‐body and decreases toward the tail (Figure 6b). CBL also decreases toward the tail, particularly in benthic species (Figure 6c).

### 3.3 Diversity of soft tissue anatomy

**FIGURE 7:**
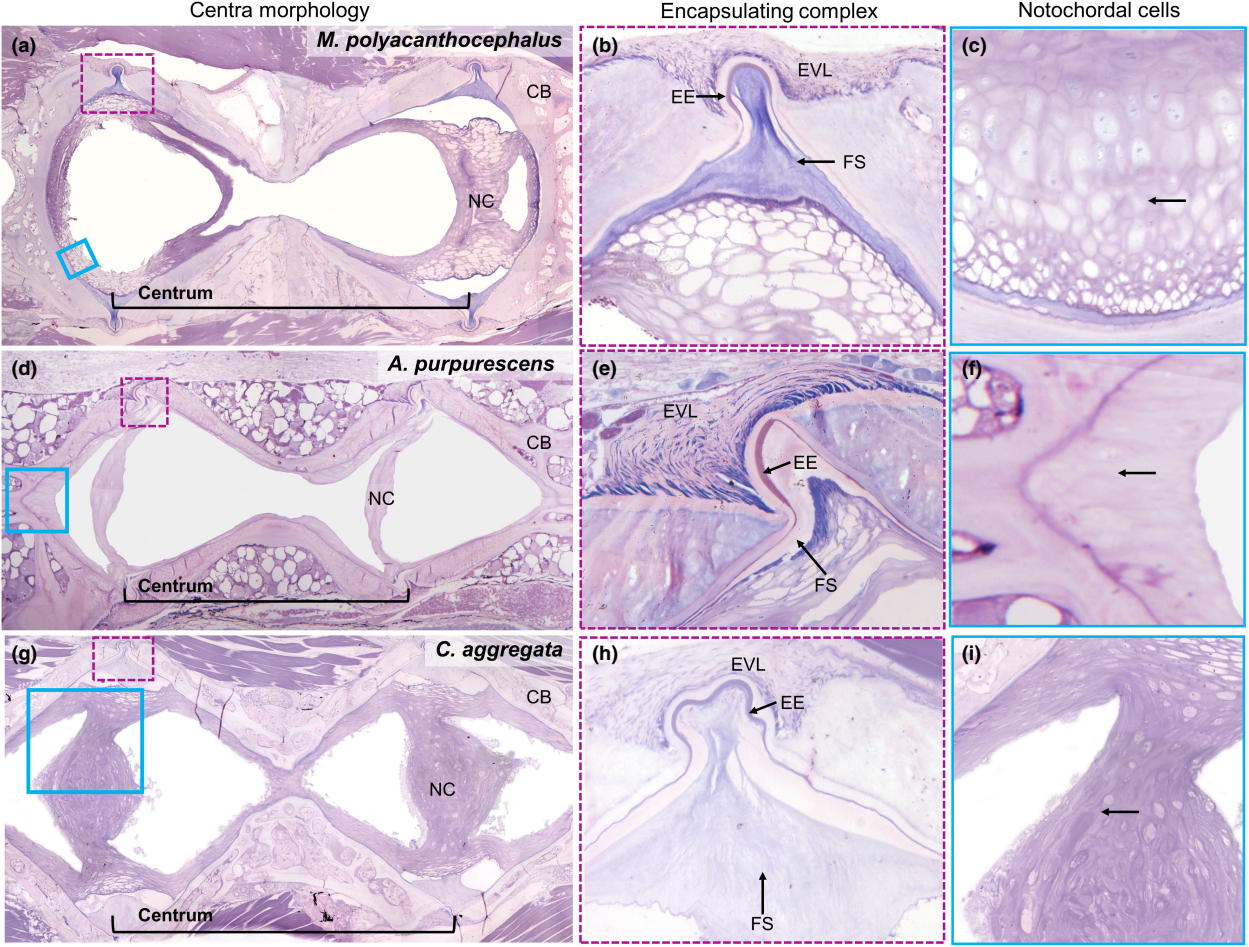
Histological sections from three representative species show differences in soft tissue anatomy. Histological sections from a benthic (*Myoxocephalus polyacanthocephalus*, a–c), demersal (*Anoplarchus purpurescens*, d, e), and pelagic (*Cymatogaster aggregata*, g–i) species. Vertebrae are oriented so that anterior is to the left and posterior to the right. The first column (a, d, g) shows an entire centrum that is composed of cortical bone (CB), filled with notochordal material (NC), and joined to adjacent centra by fibrous and sponge tissues (dashed purple box). The second column (b, e, h) highlights the encapsulating complex that holds the only elastic material and includes the external intervertebral ligament (EVL), elastica externa (EE), and fibrous sheath (FS). The third column (c, f, i) highlights the different shapes of the notochordal cells in the three species. The purple dashed box and the blue box indicate the locations of images shown in the second and third columns, respectively. *Note that panel (c) is from a separate section.

To examine the underlying tissues more thoroughly, we also imaged tissue structures in vertebral centra using histological methods (Figure 7). We chose three representative fish species (*M. polyacanthocephalus*, benthic, Figure 7a–c; *A. purpurescens*, demersal, Figure 7d–f; *C. aggregata*, pelagic, Figure 7g–i) based on availability in the Friday Harbor Labs ichthyological collection. In all species, the amphicoelous vertebral centra are composed of cortical bone (CB), with two fluid vacuoles, and various notochordal tissues (Figure 6). Each vertebra has a pair of encapsulating complexes (EC) located on the anterior and posterior sides of the centra. These ECs are composed of three main tissues: (1) the EVL, a membranous ligament that attaches adjacent vertebrate, (2) the EE, a thin layer composed of elastin, and (3) a collagen‐containing FS. The largest differences in soft tissue morphology were apparent in the intervertebral spaces for each species.

The volume of notochordal tissues (NC) was lowest in *A. purpurescens*, the demersal representative, whereas both *C. aggregata* (pelagic) and *M. polyacanthocephalus* (benthic) had qualitatively similar notochordal volumes. Histological sectioning revealed clearly defined notochordal foramen in all three species. In *A. purpurescens*, the foramen is filled with a relatively thin notochordal cell mass composed of elongated notochordal cells (Figure 7f) and an epithelium extending the length of the intervertebral junction. In both *C. aggregata* (pelagic) and *M. polyacanthocephalus* (benthic), we see rounded and elongated notochordal cells as part of the notochordal cell mass (Figure 7c,i). Similarly, the cortical bone of the vertebrae in pelagic and benthic representatives is much more robust than in our demersal representative.

The cellular morphology of the external intervertebral ligament was qualitatively similar across all three species but the EE and FS varies across species, and these were the two tissues responsible for changing tensile properties between vertebral elements. The FSs, sometimes referred to as the notochordal sheaths, of *C. aggregata* (pelagic, Figure 7h) and *M. polyacanthocephalus* (benthic, Figure 7b) were similar and composed primarily of acellular tissue. In *A. purpurescens* (demersal, Figure 7e), the FS was smaller and thinner, though still acellular in morphology. The EE, the only elastin‐containing tissue between vertebrae, was again most similar between the benthic and pelagic species and is notably asymmetrical in the demersal species (Figure 7).

## 4 DISCUSSION

We have shown that fish species that reside in benthic, pelagic, and demersal habitats have vertebrae with significantly different morphology. Mean notochordal foramen diameter and posterior cone angle were significantly different for fishes from the different habitats. CBL and the anterior and posterior cone diameters not only tended to be smaller in more elongate fishes but also showed some significant differences across habitats. In general, fishes from pelagic habitats tended to have shallower cones and narrower foramina than fishes from the other habitats, while benthic species have wider cones (Figures 4a,b and Figure 5). The morphology of the soft tissue around and within the vertebral centra was different in our representative demersal species (*Anoplarchus purpurescens*) then the pelagic and benthic representatives (Figure  7).

On average, this means that pelagic fishes have less space for notochordal material and other flexible tissues, probably making their backbones passively stiffer. In both physical models and tests on excised vertebral columns, these shallower cones and narrower foramina are associated with stiffer intervertebral joints (Donatelli et al., 2021). The connection between body mechanics and swimming performance is still being examined, but in robotic models, adding stiff vertebral centra and reducing the amount of flexible notochordal material increased the stiffness of an artificial vertebral column and led to higher speed steady swimming (Long et al., 2011). More recently, increasing the stiffness of a tuna robot increased its swimming speed, up to an optimum, but then only decreased the speed slightly as stiffness increased further (Zhong et al., 2021). Thus, we suggest that these differences result in pelagic fishes having overall stiffer backbones, which may help them to swim continuously (Lauder, 2015). Conversely, demersal and benthic species have larger cones and relatively more space for notochordal material, making their backbones more flexible. Many benthic species, such as sculpins, are ambush predators, catching prey with high accelerations that are often accompanied by large body curvature, which could be facilitated by the larger cone angles in their vertebrae. Additionally, their more flexible vertebral columns may allow them more behavioral flexibility. For example, a greater range of speeds are possible if an animal can modulate its body stiffness (Wolf et al., 2020; Zhong et al., 2021). The more passively flexible bodies of demersal and benthic fishes may thus give them more active control of their body stiffness leading to a greater diversity of swimming modes. As these fish range from ambush predators like the sculpins to burrowers like flatfish, greater control of body stiffness would be advantageous.

Histology on three representative species revealed the diversity of soft tissues across depths, and while our examples represent specific species, they do point out key traits that are likely beneficial for their specific habitats. Our representative pelagic and benthic species have more robust skeletal structure and more notochordal material then the demersal species (Figure 6). Our pelagic representative, *Cymatogaster aggregata*, spends most of its time in the water column, swimming for prolonged periods of time, migrating, and avoiding predators. A robust vertebral column may contribute to an increase in vertebral stiffness that lends to more efficient continuous swimming—a beneficial trait for pelagic species. On the other hand, in benthic fishes, the more robust skeletal structure (Figure 6), plus the overall greater posterior cone angle (Figure 4), may result in a vertebral column that is passively more flexible than the pelagic species, but can be selectively stiffened by contractions of the surrounding muscle. This would be ideal for bursts of acceleration to escape predators or catch prey – behavior commonly seen in *Myoxocephalus polyacanthocephalus* (our histological representative for benthic environments). Demersal fish, such as *Anoplarchus purperescens*, appear to have characteristics that overlap both pelagic and benthic species, particularly an intermediate posterior cone angle (Figure 4). Many species of stichaeids, the family that includes *A. purperescens*, swim through several types of complex environments, including burrowing into the sediment, and while they are constantly moving these elongate fishes are often after sedentary prey. The combination of a reduction in notochordal material and a less robust skeletal morphology (Figure 6) perhaps point to a generalized vertebral shape for a variety of locomotor modes such as navigating the seafloor, swimming up into the water column, burrowing, and other complex behaviors.

In our data set, internal vertebral morphology is related to body shape, but in a complex way. CBL (as normalized to overall body length) was shorter in more elongate fishes (higher fineness), reflecting the fact that these species often have more vertebrae than less elongate species (Ward & Brainerd, 2007). Anterior and posterior cone diameters were also smaller in elongate fishes (Figure 4d–f, Table 4). These parameters were significantly different across habitats, with benthic and pelagic fishes relatively close to one another. Demersal species had shorter centra and smaller diameter cones, on average, but also had a wider variation in these parameters, likely reflecting the high variability in behavior in demersal species. Other parameters, including posterior cone angle and foramen diameter, did not correlate significantly with fineness, but were significantly different in different habitats.

Pelagic species were significantly more elongate than benthic species (Figure 3), similar to what has been observed in previous studies (Burress et al., 2017; Cooper et al., 2010; Friedman et al., 2020; Gerry et al., 2011; Hollingsworth et al., 2013; Tavera et al., 2018; Willacker et al., 2010). For pelagic fishes with narrow and shallow bodies, there is less skin and muscle in the body's cross‐section than for benthic fishes. Thus, a smaller foramen diameter and larger cone angle of an individual vertebra may have a higher contribution to the mechanical properties of the whole body because they represent a larger fraction of the cross‐section. This might mean that the whole‐body properties of pelagic fishes are more influenced by the mechanics of the backbone than those of benthic fishes.

The pattern of vertebral shape along the body differs for fishes from different habitats (Figure 6). For all fishes, the foramen diameter has a maximum at some point along the body, and then tends to get smaller in more posterior and more anterior vertebrae. The foramen diameter tends to be more uniform along the body for pelagic fishes. CBL also varies along the body. Pelagic species again have vertebrae with relatively uniform length, where demersal and benthic species tend to have longer vertebrae more anteriorly and shorter vertebrae more posteriorly. The functional significance of these changes in vertebral shape along the body is hard to interpret. In benthic and demersal fishes, it may be that the anterior vertebral column is more flexible than the posterior portion because the space for flexible materials is smaller near the tail, whereas in pelagic fishes, the mechanical properties may be more uniform along the body. Though a stiffer body leads to faster speeds at low undulation frequencies, bodies that are more flexible toward the tail result in faster speeds at high frequencies and are also more efficient (Lucas et al., 2015). Mapping changes in vertebral morphology may therefore have implications in our understanding of locomotor efficiency, though other factors likely play a role in whole‐body stiffness, like the mechanics of the skin and muscle as well as the shape of the body, which tends to vary in thickness particularly in benthic and demersal fishes (Friedman et al., 2020).

In contrast, demersal and benthic fishes tend to spend more time maneuvering around complex obstacles like coral reefs (Larouche et al., 2020). This often requires the body to bend with much higher curvature than during steady swimming. A more flexible backbone may permit these high curvatures. In addition, larger cone angles may allow the intervertebral joints to flex to a higher angle before the bone of the vertebra begins to limit bending (Nowroozi & Brainerd, 2013). Rapid turns or those with a small turning radius also typically require high curvature in the anterior body. For example, consider C‐start escape responses, which require much higher anterior body curvature than steady swimming (Domenici & Blake, 1997; Gerry et al., 2012). This anterior curvature may be facilitated by the differences in vertebral morphology from anterior to posterior in benthic and demersal fishes (Figure 6).

Overall, the differences we observed are likely the result of selective processes that operate on the entire body as a functional unit, not selection for specific parameters that we measured. Moreover, body shape and vertebral morphology are likely to be coupled developmentally. The patterns we observe may therefore be a consequence of some other functional specialization (e.g. Gould & Lewontin, 1979), perhaps for body shape, but they are nevertheless relevant because they help distinguish species from different environments and may point to broader functional differences in those species.

## 5 CONCLUSION

The internal morphology of the vertebrae of actinopterygian fishes differs across species with several overlapping patterns in fishes classified as benthic, demersal, and pelagic. These morphological differences were especially distinct between species from benthic and pelagic habitat groups. Overall, the differences seem to be consistent with commonly observed behavioral differences between benthic and pelagic fishes, where benthic fishes tend to maneuver more around complex substrates and do not tend to swim steadily, and pelagic fishes swim constantly but do not need to maneuver as much as benthic or demersal fishes. This work adds to the body of literature (Donatelli et al., 2021; Porter et al., 2007, 2014; Porter & Long, 2010) suggesting that creating a model of vertebral morphology in fishes has implications in modeling kinematic diversity. This will be especially powerful in developing an understanding of the kinematic patterns of extinct fishes or extant fishes that are difficult to keep.

## AUTHOR CONTRIBUTIONS

**Dana Baxter:** Data curation (lead); formal analysis (equal); investigation (lead); software (supporting); visualization (supporting); writing – original draft (lead); writing – review and editing (equal). **Karly E. Cohen:** Investigation (supporting); visualization (supporting); writing – review and editing (equal). **Cassandra M. Donatelli:** Conceptualization (lead); investigation (supporting); supervision (supporting); visualization (supporting); writing – review and editing (equal). **Eric D. Tytell:** Conceptualization (supporting); formal analysis (equal); investigation (supporting); project administration (lead); software (lead); supervision (lead); visualization (lead); writing – review and editing (equal).

## FUNDING INFORMATION

This work was supported by NSF grant IOS 1652582 to E.D.T.

## CONFLICT OF INTEREST

The authors declare that they have no competing interests.

### OPEN RESEARCH BADGES

This article has earned an Open Data badge for making publicly available the digitally‐shareable data necessary to reproduce the reported results. The data is available at https://tytell.github.io/BaxterVertEvol/.

## Supporting information


Table S1

## Data Availability

All data and analysis scripts are available with DOI https://doi.org/10.25833/772t‐cw09. The most recent version of the analysis scripts is also available at GitHub (https://github.com/tytell/BaxterVertEvol).

## References

[ece39499-bib-0001] Adams, D. C. , & Collyer, M. L. (2018). Phylogenetic ANOVA: Group‐clade aggregation, biological challenges, and a refined permutation procedure. Evolution, 72, 1204–1215. 29682730 10.1111/evo.13492

[ece39499-bib-0002] Akanyeti, O. , Putney, J. , Yanagitsuru, Y. R. , Lauder, G. V. , Stewart, W. J. , & Liao, J. C. (2017). Accelerating fishes increase propulsive efficiency by modulating vortex ring geometry. Proceedings of the National Academy of Sciences of the United States of America, 114, 13828–13833. 29229818 10.1073/pnas.1705968115 PMC5748167

[ece39499-bib-0003] Allen, G. R. , Midgley, S. H. , & Allen, M. (2002). Field guide to the freshwater fishes of Australia. CSIRO Publishing.

[ece39499-bib-0004] Bailey, R. G. (1994). Guide to the fishes of the River Nile in the Republic of the Sudan. Journal of Natural History, 28, 937–970.

[ece39499-bib-0005] Baken, E. K. , Collyer, M. L. , Kaliontzopoulou, A. , & Adams, D. C. (2021). Geomorph v4.0 and gmShiny: Enhanced analytics and a new graphical interface for a comprehensive morphometric experience. Methods in Ecology and Evolution, 12, 2355–2363.

[ece39499-bib-0006] Basolo, A. L. (1990). Female preference for male sword length in the green swordtail, *Xiphophorus helleri* (Pisces: Poeciliidae). Animal Behaviour, 40, 332–338.

[ece39499-bib-0007] Blake, R. W. , Law, T. C. , Chan, K. H. S. , & Li, J. F. Z. (2005). Comparison of the prolonged swimming performances of closely related, morphologically distinct three‐spined sticklebacks *Gasterosteus* spp. Journal of Fish Biology, 67, 834–848.

[ece39499-bib-0008] Burress, E. D. , Holcomb, J. M. , Tan, M. , & Armbruster, J. W. (2017). Ecological diversification associated with the benthic‐to‐pelagic transition by north American minnows. Journal of Evolutionary Biology, 30, 549–560. 27925684 10.1111/jeb.13024

[ece39499-bib-0009] Collyer, M. L. , & Adams, D. C. (2018). RRPP: An r package for fitting linear models to high‐dimensional data using residual randomization. Methods in Ecology and Evolution, 9, 1772–1779.

[ece39499-bib-0010] Cooper, W. J. , Parsons, K. , McIntyre, A. , Kern, B. , McGee‐Moore, A. , & Albertson, R. C. (2010). Bentho‐pelagic divergence of cichlid feeding architecture was prodigious and consistent during multiple adaptive radiations within African Rift‐Lakes. PLoS One, 5, e9551. 20221400 10.1371/journal.pone.0009551 PMC2833203

[ece39499-bib-0011] Domenici, P. , & Blake, R. W. (1997). The kinematics and performance of fish fast‐start swimming. The Journal of Experimental Biology, 200, 1165–1178. 9319004 10.1242/jeb.200.8.1165

[ece39499-bib-0012] Donatelli, C. M. , Roberts, A. S. , Scott, E. , DeSmith, K. , Summers, D. , Abu‐Bader, L. , Baxter, D. , Standen, E. M. , Porter, M. E. , Summers, A. P. , & Tytell, E. D. (2021). Foretelling the flex—Vertebral shape predicts behavior and ecology of fishes. Integrative and Comparative Biology, 61, 414–426. 34048550 10.1093/icb/icab110

[ece39499-bib-0013] Ehlinger, T. J. , & Wilson, D. S. (2006). Complex foraging polymorphism in bluegill sunfish. Proceedings of the National Academy of Sciences of the United States of America, 85, 1878–1882. 10.1073/pnas.85.6.1878 PMC279884 16578831

[ece39499-bib-0014] Ellerby, D. J. , & Gerry, S. P. (2011). Sympatric divergence and performance trade‐offs of bluegill ecomorphs. Evolutionary Biology, 38, 422–433.

[ece39499-bib-0015] Fine, M. L. , Horn, M. H. , Cox, B. , & Marshall, N. B. (1987). *Acanthonus armatus*, a deep‐sea teleost fish with a minute brain and large ears. Proceedings of the Royal Society of London ‐ Series B: Biological Sciences, 230, 257–265. 2884671 10.1098/rspb.1987.0018

[ece39499-bib-0016] Friedman, S. T. , Price, S. A. , Corn, K. A. , Larouche, O. , Martinez, C. M. , & Wainwright, P. C. (2020). Body shape diversification along the benthic–pelagic axis in marine fishes. Proceedings of the Royal Society B: Biological Sciences, 287, 20201053. 10.1098/rspb.2020.1053 PMC7423681 32693721

[ece39499-bib-0017] Gerry, S. P. , Robbins, A. , & Ellerby, D. J. (2012). Variation in fast‐start performance within a population of polyphenic bluegill (*Lepomis macrochirus*). Physiological and Biochemical Zoology, 85, 694–703. 23099466 10.1086/667593

[ece39499-bib-0018] Gerry, S. P. , Wang, J. , & Ellerby, D. J. (2011). A new approach to quantifying morphological variation in bluegill *Lepomis macrochirus* . Journal of Fish Biology, 78, 1023–1034. 21463305 10.1111/j.1095-8649.2011.02911.x

[ece39499-bib-0019] Gilbert, C. R. , & Williams, J. D. (2002). National audubon society field guide to fishes: North America (2nd ed.). Knopf.

[ece39499-bib-0020] Gould, S. J. , & Lewontin, R. C. (1979). The spandrels of San Marco and the Panglossian paradigm: A critique of the adaptationist programme. Proceedings of the Royal Society B: Biological Sciences, 205, 581–598. 42062 10.1098/rspb.1979.0086

[ece39499-bib-0021] Gower, J. C. (1975). Generalized procrustes analysis. Psychometrika, 40, 33–51.

[ece39499-bib-0022] Hollingsworth, P. R., Jr. , Simons, A. M. , Fordyce, J. A. , & Hulsey, C. D. (2013). Explosive diversification following a benthic to pelagic shift in freshwater fishes. BMC Evolutionary Biology, 13, 272. 24341464 10.1186/1471-2148-13-272 PMC3880099

[ece39499-bib-0023] Hulsey, C. D. , Roberts, R. J. , Loh, Y.‐H. E. , Rupp, M. F. , & Streelman, J. T. (2013). Lake Malawi cichlid evolution along a benthic/limnetic axis. Ecology and Evolution, 3, 2262–2272. 23919168 10.1002/ece3.633 PMC3728963

[ece39499-bib-0024] Jaafar, Z. , Hajisamae, S. , Chou, L. M. , & Yatiman, Y. (2004). Community structure of coastal fishes in relation to heavily impacted human modified habitats. Hydrobiologia, 511, 113–123.

[ece39499-bib-0025] Koob, T. J. , & Long, J. H. (2000). The vertebrate body axis: Evolution and mechanical function. American Zoologist, 40, 1–18.

[ece39499-bib-0026] Kusche, H. , Recknagel, H. , Elmer, K. R. , & Meyer, A. (2014). Crater lake cichlids individually specialize along the benthic‐limnetic axis. Ecology and Evolution, 4, 1127–1139. 24772288 10.1002/ece3.1015 PMC3997327

[ece39499-bib-0027] Laerm, J. (1976). The development, function, and design of amphicoelous vertebrae in teleost fishes. Zoological Journal of the Linnean Society, 58, 237–254.

[ece39499-bib-0028] Larouche, O. , Benton, B. , Corn, K. A. , Friedman, S. T. , Gross, D. , Iwan, M. , Kessler, B. , Martinez, C. M. , Rodriguez, S. , Whelpley, H. , Wainwright, P. C. , & Price, S. A. (2020). Reef‐associated fishes have more maneuverable body shapes at a macroevolutionary scale. Coral Reefs, 39, 1427–1439.

[ece39499-bib-0029] Lauder, G. V. (2015). Fish locomotion: Recent advances and new directions. Annual Review of Marine Science, 7, 521–545. 10.1146/annurev-marine-010814-015614 25251278

[ece39499-bib-0030] Link, J. S. , Bolles, K. , & Milliken, C. G. (2002). The feeding ecology of flatfish in the Northwest Atlantic. Journal of Northwest Atlantic Fishery Science, 30, 1–17.

[ece39499-bib-0031] Long, J. H. (1992). Stiffness and damping forces in the intervertebral joints of blue marlin (*Makaira nigricans*). The Journal of Experimental Biology, 162, 131–155.

[ece39499-bib-0032] Long, J. H. , Koob‐Emunds, M. , & Koob, T. J. (2004). The mechanical consequences of vertebral centra. Bulletin of the Mount Desert Island Biological Laboratory, 43, 99–101.

[ece39499-bib-0033] Long, J. H. , Krenitsky, N. M. , Roberts, S. F. , Hirokawa, J. , de Leeuw, J. , & Porter, M. E. (2011). Testing biomimetic structures in bioinspired robots: How vertebrae control the stiffness of the body and the behavior of fish‐like swimmers. Integrative and Comparative Biology, 51, 158–175. 21576117 10.1093/icb/icr020

[ece39499-bib-0034] Lowry, D. , Wintzer, A. P. , Matott, M. P. , Whitenack, L. B. , Huber, D. R. , Dean, M. , & Motta, P. J. (2005). Aerial and aquatic feeding in the silver arawana, *Osteoglossum bicirrhosum* . Environmental Biology of Fishes, 73, 453–462.

[ece39499-bib-0035] Lucas, K. N. , Thornycroft, P. J. , Gemmell, B. J. , Colin, S. P. , Costello, J. H. , & Lauder, G. V. (2015). Effects of non‐uniform stiffness on the swimming performance of a passively‐flexing, fish‐like foil model. Bioinspiration & Biomimetics, 10, 056019. 26447541 10.1088/1748-3190/10/5/056019

[ece39499-bib-0036] Magid, A. M. A. (1967). Respiration of air by the primitive fish *Polypterus senegalus* . Nature, 215, 1096–1097.

[ece39499-bib-0037] Matsui, T. , & Rosenblatt, R. H. (1987). Review of the deep‐sea fish family Platytroctidae (Pisces: Salmoniformes). Bulletin of the Scripps Institution of Oceanography, 26, 1–159.

[ece39499-bib-0038] McGinnis, S. M. , & Alcorn, D. (2006). Trout and salmon (Salmonidae). In Field guide to freshwater fishes of California: Revised edition (pp. 147–195). University of California Press.

[ece39499-bib-0039] Mérigoux, S. , Ponton, D. , & de Mérona, B. (1998). Fish richness and species‐habitat relationships in two coastal streams of French Guiana, South America. Environmental Biology of Fishes, 51, 25–39.

[ece39499-bib-0040] Muschick, M. , Indermaur, A. , & Salzburger, W. (2012). Convergent evolution within an adaptive radiation of cichlid fishes. Current Biology, 22, 2362–2368. 23159601 10.1016/j.cub.2012.10.048

[ece39499-bib-0041] Nowroozi, B. N. , & Brainerd, E. L. (2012). Regional variation in the mechanical properties of the vertebral column during lateral bending in *Morone saxatilis* . Journal of the Royal Society Interface, 9, 2667–2679. 22552920 10.1098/rsif.2012.0153 PMC3427503

[ece39499-bib-0042] Nowroozi, B. N. , & Brainerd, E. L. (2013). X‐ray motion analysis of the vertebral column during the startle response in striped bass, *Morone saxatilis* . The Journal of Experimental Biology, 216, 2833–2842. 23842627 10.1242/jeb.085118

[ece39499-bib-0043] Nowroozi, B. N. , & Brainerd, E. L. (2014). Importance of mechanics and kinematics in determining the stiffness contribution of the vertebral column during body‐caudal‐fin swimming in fishes. Zoology, 117, 28–35. 24374037 10.1016/j.zool.2013.10.003

[ece39499-bib-0044] Page, L. M. , Burr, B. M. , Society, N. A. , Federation, N. W. , & Institute, R. T. P. (1991). Field guide to freshwater fishes: North America, North of Mexico, Special edition. Houghton Mifflin Harcourt.

[ece39499-bib-0045] Pearcy, W. G. , Stein, D. L. , & Carney, R. S. (1982). The deep‐sea benthic fish fauna of the northeastern Pacific Ocean on Cascadia and Tufts abyssal plains and adjoining continental slopes. Biological Oceanography, 1, 375–428.

[ece39499-bib-0046] Phomikong, P. , Fukushima, M. , Sricharoendham, B. , Nohara, S. , & Jutagate, T. (2015). Diversity and community structure of fishes in the regulated versus unregulated tributaries of the Mekong River. River Research and Applications, 31, 1262–1275.

[ece39499-bib-0047] Pietsch, T. W. , & Orr, J. W. (2015). Fishes of the Salish Sea: A compilation and distributional analysis. United States Department of Commerce, National Oceanic and Atmospheric Administration, National Marine Fisheries Service, Scientific Publications Office.

[ece39499-bib-0048] Porter, M. E. , Diaz, C., Jr. , Sturm, J. J. , Grotmol, S. , Summers, A. P. , & Long, J. H. (2014). Built for speed: Strain in the cartilaginous vertebral columns of sharks. Zoology, 117, 19–27. 24388493 10.1016/j.zool.2013.10.007

[ece39499-bib-0049] Porter, M. E. , Koob, T. J. , & Summers, A. P. (2007). The contribution of mineral to the material properties of vertebral cartilage from the smooth‐hound shark *Mustelus californicus* . The Journal of Experimental Biology, 210, 3319–3327. 17872985 10.1242/jeb.006189

[ece39499-bib-0050] Porter, M. E. , & Long, J. H. (2010). Vertebrae in compression: Mechanical behavior of arches and centra in the gray smooth‐hound shark (*Mustelus californicus*). Journal of Morphology, 271, 366–375. 19862836 10.1002/jmor.10803

[ece39499-bib-0051] Proctor, N. S. , & Lynch, P. J. (2011). Fish. In A field guide to the southeast coast and Gulf of Mexico (pp. 88–181). Yale University Press.

[ece39499-bib-0052] Rabosky, D. L. , Chang, J. , Title, P. O. , Cowman, P. F. , Sallan, L. , Friedman, M. , Kaschner, K. , Garilao, C. , Near, T. J. , Coll, M. , & Alfaro, M. E. (2018). An inverse latitudinal gradient in speciation rate for marine fishes. Nature, 559, 392–395. 29973726 10.1038/s41586-018-0273-1

[ece39499-bib-0053] Ribeiro, E. , Davis, A. M. , Rivero‐Vega, R. A. , Orti, G. , & Betancur‐R, R. (2018). Post‐cretaceous bursts of evolution along the benthic‐pelagic axis in marine fishes. Proceedings of the Royal Society B: Biological Sciences, 285, 20182010. 10.1098/rspb.2018.2010 PMC6304066 30963906

[ece39499-bib-0054] Robinson, B. W. , & Wilson, D. S. (1994). Character release and displacement in fishes: A neglected literature. The American Naturalist, 144, 596–627.

[ece39499-bib-0055] Rodríguez‐Olarte, D. , Mojica Corzo, J. I. , & Taphorn Baechle, D. C. (2011). Northern South America: Magdalena and Maracaibo basins. In J. S. Albert & R. Reis (Eds.), Historical biogeography of neotropical freshwater fishes (pp. 243–257). University of California Press.

[ece39499-bib-0056] Schluter, D. (1993). Adaptive radiation in sticklebacks: Size, shape, and habitat use efficiency. Ecology, 74, 699–709.

[ece39499-bib-0057] Schwalbe, M. A. B. , Boden, A. L. , Wise, T. N. , & Tytell, E. D. (2019). Red muscle activity in bluegill sunfish *Lepomis macrochirus* during forward accelerations. Scientific Reports, 9, 8088. 31147566 10.1038/s41598-019-44409-7 PMC6542830

[ece39499-bib-0058] Summers, A. P. , & Long, J. H. (2006). Skin and bones, sinew and gristle: The mechanical behavior of fish skeletal tissues. In R. E. Shadwick & G. V. Lauder (Eds.), Fish physiology (pp. 141–177). Elsevier.

[ece39499-bib-0059] Symmons, S. (1979). Notochordal and elastic components of the axial skeleton of fishes and their functions in locomotion. Journal of Zoology, 189, 157–206.

[ece39499-bib-0060] Tavera, J. , Acero, P. A. , & Wainwright, P. C. (2018). Multilocus phylogeny, divergence times, and a major role for the benthic‐to‐pelagic axis in the diversification of grunts (Haemulidae). Molecular Phylogenetics and Evolution, 121, 212–223. 29307507 10.1016/j.ympev.2017.12.032

[ece39499-bib-0061] van der Sleen, P. , & Albert, J. S. (Eds.). (2018). The fish families. In Field guide to the fishes of the Amazon, Orinoco, and Guianas (pp. 69–402). Princeton University Press.

[ece39499-bib-0062] Vašek, M. , Jarolím, O. , Čech, M. , Kubečka, J. , Peterka, J. , & Prchalová, M. (2008). The use of pelagic habitat by cyprinids in a deep riverine impoundment: Římov Reservoir, Czech Republic. Folia Zoologica, 57, 324–336.

[ece39499-bib-0063] Walker, J. (1997). Ecological morphology of lacustrine threespine stickleback *Gasterosteus aculeatus* L (Gasterosteidae) body shape. Biological Journal of the Linnean Society, 61, 3–50.

[ece39499-bib-0064] Ward, A. B. , & Brainerd, E. L. (2007). Evolution of axial patterning in elongate fishes. Biological Journal of the Linnean Society, 90, 97–116.

[ece39499-bib-0065] Willacker, J. J. , Von Hippel, F. A. , Wilton, P. R. , & Walton, K. M. (2010). Classification of threespine stickleback along the benthic‐limnetic axis. Biological Journal of the Linnean Society, 101, 595–608. 21221422 10.1111/j.1095-8312.2010.01531.x PMC3017379

[ece39499-bib-0066] Wolf, Z. , Jusufi, A. , Vogt, D. M. , & Lauder, G. V. (2020). Fish‐like aquatic propulsion studied using a pneumatically‐actuated soft‐robotic model. Bioinspiration & Biomimetics, 15, 046008. 32330908 10.1088/1748-3190/ab8d0f

[ece39499-bib-0067] Zhong, Q. , Zhu, J. , Fish, F. E. , Kerr, S. J. , Downs, A. M. , Bart‐Smith, H. , & Quinn, D. B. (2021). Tunable stiffness enables fast and efficient swimming in fish‐like robots. Science Robotics, 6, eabe4088. 34380755 10.1126/scirobotics.abe4088

